# Malignant Pleural Effusions—A Review of Current Guidelines and Practices

**DOI:** 10.3390/jcm10235535

**Published:** 2021-11-26

**Authors:** Prarthna Chandar Kulandaisamy, Sakthidev Kulandaisamy, Daniel Kramer, Christopher Mcgrath

**Affiliations:** Department of Pulmonary, Allergy and Critical Care, Thomas Jefferson University Hospital, Philadelphia, PA 19107, USA; sakthi484@gmail.com (S.K.); Daniel.kramer@jefferson.edu (D.K.); Christopher.mcgrath@jefferson.edu (C.M.)

**Keywords:** malignant pleural effusion, lung cancer, pleurodesis, indwelling pleural catheter, thoracentesis

## Abstract

Malignant pleural effusion (MPE) occurs in 15% of all cancer patients and usually portends poor prognosis while also serving to limit the patient’s quality of life. Palliation of symptoms has been the goal for the management of these effusions while keeping the patient’s hospital stay to a minimum. Traditionally, this has been achieved by chest tube drainage followed by the instillation of sclerosing agents, such as talc, in the pleural space. A recent increase in evidence for the effectiveness and convenience of indwelling pleural catheters has changed the management of MPE, which is reflected in the guidelines released by the American Thoracic Society as well their European Counterpart (ERS/BTS). In this article, we aim to review the current management practices and guidelines for MPE.

## 1. Introduction

Malignant pleural effusion (MPE) is the second most common cause of pleural exudate and affects 15% of all patients with cancer [1]. MPE accounts for greater than 125,000 admissions per year in the United States, with costs of around $5 billion for inpatient charges alone. It usually indicates an advanced stage or metastatic disease and hence portends a poor prognosis. The average life expectancy of patients presenting with MPE is 3–12 months depending on the underlying tumor type and patient comorbidities [2]. Lung cancer, breast cancer, and lymphoma contribute to the majority of these effusions, followed by gynecological malignancies and malignant mesothelioma [3]. Patients frequently experience debilitating dyspnea and other symptoms which severely compromise their quality of life. Various therapies are currently available for the management of these effusions. The management of MPE is challenging and is mainly focused on the relief of symptoms and the improvement of the patients’ quality of life. The therapy needs to be tailored to individual patients taking into account their preferences, life expectancy, affordability, presence of trapped lungs, resources available, and the experience of the treatment team. In this review, we will discuss the recent guidelines and practices in the management of MPE.

## 2. Clinical Presentation

Dyspnea is the most common symptom which occurs in almost all patients with MPE [4]. Even small size effusions can cause significant dyspnea, and the severity of breathlessness often correlates poorly with the size of the effusion. Factors likely contributing to this include: a loss of functional lung tissues due to atelectasis secondary to the MPE; decreased chest wall compliance; chest wall expansion; displacement of the diaphragm; and mediastinal shift. Even small effusions can cause significant dyspnea and patients experience relief of symptoms with drainage of as low as 200–300 cc pleural fluid due to the deflation of the thoracic cage and associated improvement in the respiratory muscle function. Roughly 60% of the patients also experience dull chest pain [5,6]. Another nonspecific and common complaint is a cough. Patients can also experience constitutional symptoms such as fever, loss of weight, loss of appetite, night sweats, and fatigue. Patients may also experience symptoms related to their primary cancers though in many cases, MPE presents itself as the first sign of disease.

## 3. Diagnosis

### 3.1. Imaging

Almost all radiological procedures can help us identify MPE. As little as 50 mL of fluid can be identified in a lateral view of the chest x-ray, which usually presents as blunting of the costophrenic angle. Ultrasound of the chest (USG) is more sensitive and may help identify any pleural metastasis as well as an assessment of pleural thickening [7]. It can also help characterize the pleural fluid and identify septations and loculations. Echogenic swirling pattern is characterized by numerous free-floating echogenic particles swirling in the pleural cavity during respiratory movements. Heartbeat is another pointer towards possible MPE. USG is also an important tool in the therapeutic aspect, as it helps guide thoracentesis and the placement of chest tubes. Following the aspiration of pleural fluid, USG also helps in the quick assessment of the re-expansion of the underlying lung parenchyma as well as the identification of any pneumothorax [8].

CT chest scans are highly sensitive and also give additional information on underlying lung pathology, pleural thickening, pleural nodularity, the presence of loculations, and other characters which can point towards a malignant etiology of the pleural effusion [9]. Several studies are underway in determining CT features that will help in the diagnosis of MPE. Other imaging modalities that can be helpful include MRI and PET-CT, though the availability and cost may prohibit their wide use.

### 3.2. Pathological Diagnosis

MPE occurs due to tumor metastasis to the pleura/pleural space either by direct invasion, hematogenous, or lymphangitic spread. The diagnosis of MPE requires the presence of tumor cells in the pleural fluid or evidence of tumor presence in a pleural biopsy. A USG guided thoracentesis has a diagnostic yield of 60% for the first aspiration and increases to 70–75% following a second aspiration but plateaus after [5]. The sensitivity varies per underlying tumor type. The sensitivity of pleural fluid for diagnosis of malignant mesothelioma is even less than the stated average [10]. Blind percutaneous pleural biopsy has less sensitivity compared to image-guided (CT or USG) biopsy, which can have a yield as high as thoracoscopic parietal pleural biopsy (95%) [11]. Thoracoscopic biopsy can be done either by medical thoracoscopy or video-assisted thoracoscopy (VATS).

The pleural fluid is usually exudative by Light’s criteria. Light’s criteria help to differentiate an exudative effusion from a transudate and is helpful towards determining the etiology of the pleural effusion. Effusions are considered exudative if it meets any of the following criteria: (1) ratio of pleura fluid protein to serum protein is greater than 0.5, (2) The pleural fluid lactate dehydrogenase (LDH) to serum LDH ratio is greater than 0.6, (3) pleural fluid LDH is greater than 2/3 of the upper limit of normal serum LDH [12]. It has been reported that 60 mL of pleural fluid is usually adequate for diagnosis. However, the use of pleural fluid for molecular sequencing, especially in lung cancer treatment, is questionable as a large percentage of them do not demonstrate adequate cellularity for genomic studies. With improvement in molecular and tumor marker testing, this scenario will likely change in the future.

## 4. Management of MPE

A small percentage of patients with MPE remain asymptomatic and hence can be managed by observation alone [1]. The main goal for the management of symptomatic MPE is the alleviation of dyspnea and improving their quality of life by the least invasive and minimal number of procedures.

Thoracentesis is the first step in the management of symptomatic MPE. For patients with malignancies responsive to cancer-directed treatment, definitive intervention beyond this may not be necessary. Thoracentesis can be safely done in an outpatient setting as well as in the hospital. The service is offered by a wide range of specialists—pulmonologists, thoracic surgeons, interventional radiologists, emergency medicine physicians, etc. Local institutional practices and patient presentations determine which service performs the procedure. Large volume thoracentesis can be done guided by pleural manometry and with USG guidance for assessing the patient’s response to the removal of fluid. Although manometry though can be useful, is not widely used as it can be cumbersome and not readily available at all institutions. Studies have failed to establish standardized guidelines with regard to the use of manometry.

Thoracentesis also helps to identify patients with trapped lung, which can help determine further definitive interventions. Trapped lung refers to the phenomenon of the unexpandable lung, where there is an impediment to the normal apposition between the parietal and visceral pleura. The main mechanism is due to the formation of a fibrous layer along the visceral pleural surface due to local pleural pathology, which prevents lung expansion [13]. This can be assessed post procedurally after the drainage of the pleural fluid either by manometry or diagnostic imaging such as a chest X-ray/USG.

**Figure 1:**
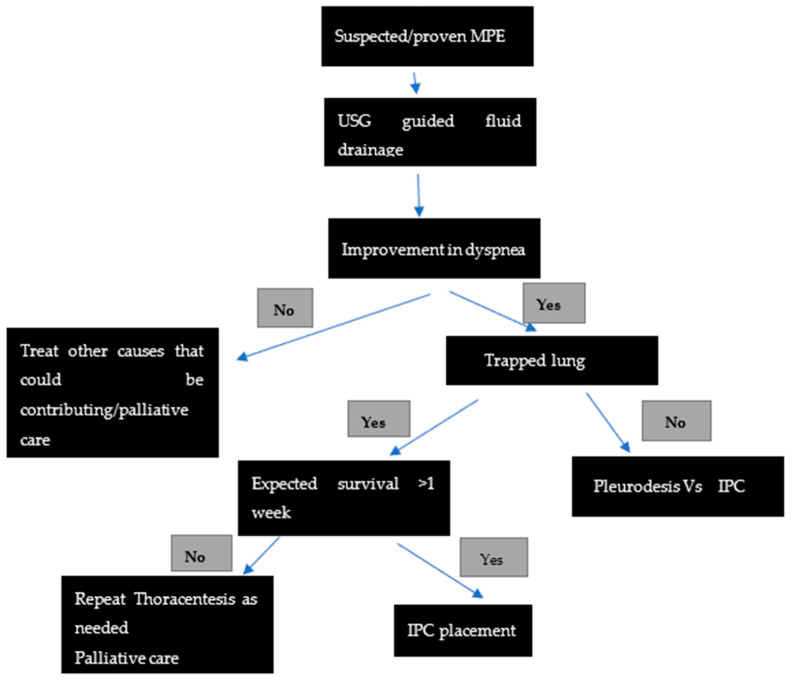
Algorithm suggested for the management of suspected/proven recurrent malignant pleural effusion.

For patients with recurrent MPE who experienced relief of dyspnea following thoracentesis, a variety of interventions are available—repeated thoracentesis, pleural space drainage with pleurodesis, insertion of an indwelling pleural catheter (IPC), and surgery. We have included a table extrapolating the current ATS guidelines on management of MPE [Table 1] as well as the practice algorithm at our institution (Figure 1). The choice of therapy should be made on a case-by-case basis taking into account the patients’ preference, affordability, quality of life, expected life expectancy, underlying tumor type, presence or absence of trapped lung, and local practices. Some tools have been validated to assess the risk of mortality, such as the LENT score (scoring based on pleural fluid LDH, ECOG performance scale, Neutrophil/lymphocyte ratio and tumor type), and can be helpful in deciding the therapy [14].

### 4.1. Thoracic Drainage and Pleurodesis

For patients with symptomatic, recurrent MPE demonstrating expandable lung, placement of chest tube for drainage, and instillation of sclerosing agents in the pleural space are recommended as the preferred definitive management. Small-bore chest tubes (10–14 French) are equally effective for this purpose compared to large-bore catheters and are recommended by British Thoracic Society Guidelines [15]. Pleurodesis involves the instillation of sclerosing agents in the pleural space to promote adhesions between the visceral and parietal pleura, thereby obliterating the space and preventing further accumulation of the pleural fluid. Various agents have been used for this purpose-talc, tetracyclines, bleomycin, Corynebacterium parvum, mitomycin, iodopovidine, etc. Talc is the most widely used and has proved to be the most efficacious [16]. Talc slurry instilled via a chest tube is equally effective as thoracoscopic talc poudrage [17]. However, highly effective talc as a sclerosant can cause significant pain in patients due to acute pleurisy. Other complications associated with talc pleurodesis include fever, acute pneumonitis, acute respiratory distress syndrome, and empyema. Studies have revealed that serious complications such as ARDS occur when small particle/non-graded talc (<15 microns) is used, likely due to high systemic absorption of talc [18].

Though highly effective in palliation of symptoms, pleurodesis involves inpatient admission of the patients and can be expensive, time-consuming, and inconvenient to the patients. This method also cannot be applied in patients with trapped lung physiology.

### 4.2. A Thoracoscopy with Pleurodesis

Thoracoscopy is a procedure that allows the visualization of the pleural surfaces and serves both as a diagnostic tool as well as definite management therapy of MPE. Thoracoscopy has high diagnostic yield (95%) in determining the etiology of exudative pleural effusions when compared to other modalities. By allowing the direct visualization of the pleural space, it allows for targeted biopsies of the parietal pleura. Additionally, it allows lysis of pleural adhesions, placement of chest tubes and pleurodesis by talc poudrage/mechanical abrasion of the parietal pleura [19]. Two different approaches are available for thoracoscopy—medical thoracoscopy (MT), completed by a pulmonologist, and surgical thoracoscopy (ST), completed by thoracic surgeons. Each has its own risks and benefits, and the choice of approach varies based on local practices, expertise, resources available, patient factors (ability to tolerate single lung ventilation/GA for ST), etc.

Medical thoracoscopy involves the placement of one or two trocars inserted through small ports along the midaxillary line. The procedure is then completed by either rigid thoracoscope or semi-rigid/flexible thoracoscope based on operator preference and expertise. MT is generally well tolerated and done under light/moderate sedation and local anesthesia and has a good safety profile. It can be completed on an outpatient basis without requiring the hospital admission of patients. Surgical thoracoscopy, usually carried out with video assistance (VATS), routinely involves three or more larger sized ports and is completed under general anesthesia. VATS normally require single-lung ventilation, which might limit patient selection for this approach. Though considered more expensive than MT, VATS carries the advantage of being able to offer the debridement of the fibrinous pleural peel in patients with trapped lung who are surgical candidates. VATS also has the option of being converted to an open thoracotomy procedure if needed. Thus, the selection of the thoracoscopic approach varies from patient to patient and by the institution and remains an area of debate in establishing the primary method of choice.

### 4.3. Indwelling Pleural Catheters (IPC)

IPCs are long silicone tubes that are inserted percutaneously under sterile precautions. These can be placed and managed on an outpatient basis. They have a one-way valve and maintain lung expansion by the intermittent drainage of pleural fluid. IPC’s have been shown to induce pleurodesis in 46–70% of patients by local inflammation by the tumor and IPC itself [20]. IPC’s are the primary modality of treatment in MPE with trapped lung. Multiple studies have established their effectiveness in achieving the palliation of symptoms. The AMPLE, TIME 2 trials have shown that IPCs are non-inferior to talc pleurodesis with regards to the relief of the patient’s dyspnea [21,22]. These trials showed a fewer number of hospital days, a fewer number of procedures, and a better dyspnea score at 6 months with IPC’s compared to talc pleurodesis. However, IPCs tend to have more adverse effects compared to talc pleurodesis (catheter-related infection primarily) and potential increased community care costs, especially in patients with a life expectancy of more than one year.

### 4.4. Other Interventions

For patients who failed chemical pleurodesis, and are not a candidate for IPC, surgical approaches can be considered. VATS pleurodesis is the primary surgical approach offered for patients who failed the primary modality of MPE management and who are surgical candidates. Radical or subtotal pleurectomy by decortication can be offered for patients with MPE due to malignant melanoma who are suitable for surgery and have a prolonged life expectancy. A pleuro-peritoneal shunt is also occasionally undertaken in rare circumstances [23].

### 4.5. Future Directions

Many trials are currently underway concerning the management of MPE (MESOTRAP, SWIFT, AMPLE 3, OPTIMUM, etc.), looking at various avenues for the successful treatment of a complex entity. The IPC PLUS trial showed significant success in achieving pleurodesis by the administration of talc through an IPC in an outpatient setting, paving the way for combined utilization of various modalities [24]. The search continues for the single most beneficial modality treatment that would offer the patient the least invasive, most effective, cost-efficient treatment while also having minimal adverse effects.

## 5. Conclusions

MPE is a common manifestation in patients having advanced lung cancer and other malignancies. There has been a considerable improvement in the diagnosis of MPE through newer cytologic and imaging techniques with improved methods of pleural biopsy. Despite the availability of therapies, the management of MPE is challenging and is mainly focused on symptom relief. IPC and talc pleurodesis have both been demonstrated to be equally efficacious in the management of these patients. Therapy should be tailored to the patient and should take into account the patient’s preferences, life expectancy, tumor type, lung expansibility, and the availability of local resources. Further research is needed to shed light on the existing controversies in the management of MPE.

## Figures and Tables

**Table 1:** Summary of Current recommendations of ATS/STS/STR [11].

PICO ^1^	RECOMMENDATIONS
1. In patients with known or suspected MPE should thoracic USG be used to guide pleural interventions?	Yes
2. In patients with known or suspected MPE, who are asymptomatic, should pleural drainage be performed?	No
3. Should the management of symptomatic known or suspected MPE guided by large volume thoracentesis and pleural manometry?	Yes. Manometry if pleurodesis is contemplated to assess for lung re-expansion
4. In patients with known or suspected symptomatic MPE, with expandable lung and no prior definitive treatment, should IPC or chemical pleurodesis be used as first line intervention?	Yes
5. In patients with known or suspected MPE, undergoing talc pleurodesis, should talc slurry or talc poudrage be used?	Yes, there is no difference in the efficacy between the two
6. In patients with symptomatic MPE with non-expandable lung, failed pleurodesis or loculated effusion, should IPC or chemical pleurodesis be used?	IPC is the preferred method of choice over chemical pleurodesis
7. In IPC associated infection, is catheter removal required?	Not unless infection does not improve

^1^ PICO-population, intervention, comparator outcomes format.
